# Process and outcome of prenatal care according to the primary care models: a cohort study*

**DOI:** 10.1590/1518-8345.2806.3058

**Published:** 2019-07-18

**Authors:** Renata Leite Alves de Oliveira, Anna Paula Ferrari, Cristina Maria Garcia de Lima Parada

**Affiliations:** 1 Universidade Estadual Paulista “Júlio de Mesquita Filho”, Faculdade de Medicina de Botucatu, Botucatu, SP, Brasil; 2 Bolsista da Coordenação de Aperfeiçoamento de Pessoal de Nível Superior (CAPES), Brasil

**Keywords:** Prenatal Care, Evaluation of Health Programs and Projects, Primary Health Care, Family Health Strategy, Child Health, Women's Health, Cuidado Pré-Natal, Avaliação de Programas e Projetos de Saúde, Atenção Primária à Saúde, Estratégia Saúde da Família, Saúde da Criança, Saúde da Mulher, Cuidado Pre-Natal, Evaluación de Programas y Proyectos de Salud, Atención Primaria a la Salud, Estrategia Salud de la Familia, Salud del Niño, Salud de la Mujer

## Abstract

**Objective:**

to evaluate the process and outcome indicators of the prenatal care developed in primary care, comparing traditional care models and the Family Health Strategy.

**Method:**

this is a cohort study, conducted with an intentional sample of 273 mothers/babies from the neonatal period and followed up for one year. Donabedian evaluation was adopted and data were discussed based on the Social Determination of Health. The independent variable was the care model. The dependent variables in the process evaluation were related to the quality of prenatal care and to the quality score created and the evaluation of the outcome, to the conditions of birth and the first year of life. The evaluation of the process was performed by estimating the relative risk and the evaluation of the outcome was performed by the Cox Multiple Regression Model.

**Results:**

lower income and risk of the low prenatal quality score were identified in the Family Health Units, where there were more puerperium consultation and health education actions. There was no difference in outcome indicators.

**Conclusion:**

possibly the best quality of prenatal care was able to minimize negative socioeconomic effects found in family health, so the outcome indicators were similar in both models of the primary care.

## Abstract (pt)

**Objetivo:**

avaliar indicadores de processo e resultado do cuidado pré-natal desenvolvido na atenção primária comparando os modelos de atenção tradicional e Estratégia Saúde da Família.

**Método:**

estudo de coorte, realizado com amostra intencional de 273 mães/bebês captados no período neonatal e acompanhados por um ano. Adotou-se referencial de avaliação de Donabedian e os dados foram discutidos a partir da Determinação Social da Saúde. A variável independente foi o modelo de atenção. As variáveis dependentes, na avaliação de processo, relacionavam-se à qualidade do pré-natal e ao escore de qualidade criado e, na avaliação de resultado, às condições de nascimento e primeiro ano de vida. A avaliação de processo foi realizada por estimativa do risco relativo e a de resultado, por Modelo de Regressão Múltipla de Cox.

**Resultados:**

foram identificadas menores rendas e risco de baixo escore de qualidade pré-natal nas Unidades de Saúde da Família, onde ocorreu mais consulta de puerpério e ações de educação em saúde. Não houve diferença nos indicadores de resultado.

**Conclusão:**

possivelmente, a melhor qualidade da atenção pré-natal foi capaz de minimizar efeitos socioeconômicos negativos encontrados na saúde da família, de modo que os indicadores de resultado foram semelhantes em ambos os modelos de atenção primária.

## Abstract (es)

**Objetivo:**

evaluar los indicadores de proceso y resultado del cuidado prenatal desarrollado en la atención primaria, comparando los modelos de atención tradicional y Estrategia Salud de la Familia.

**Método:**

estudio de cohorte, realizado con una muestra intencional de 273 madres/bebés captados en el período neonatal y acompañados por un año. Se adoptó un referencial de evaluación de Donabedian y los datos fueron discutidos a partir de la Determinación Social de la Salud. La variable independiente fue el modelo de atención. Las variables dependientes, en la evaluación de proceso, se relacionaban a la calidad del prenatal y al puntaje de calidad creado y en la evaluación de resultado, a las condiciones de nacimiento y primer año de vida. La evaluación de proceso fue realizada por estimativa del riesgo relativo y la de resultado, por Modelo de Regresión Múltiple de Cox.

**Resultados:**

fueron identificados menores rentas y riesgo de bajo puntaje de calidad pre-natal en las Unidades de Salud de la Familia, donde ocurrió más consulta de puerperio y acciones de educación en salud. No hubo diferencia en los indicadores de resultado.

**Conclusión:**

posiblemente, la mejor calidad de la atención prenatal fue capaz de minimizar efectos socioeconómicos negativos encontrados en la salud de la familia, de modo que los indicadores de resultado fueron semejantes en ambos modelos de atención primaria.

## Introduction

The perinatal period is among the priorities of global public policies because, despite the significant progress in reducing deaths during the last 15 years, unacceptably high numbers of maternal and neonatal deaths remain. Aiming at a world in which all pregnant women and newborns receive quality care during the pregnancy, the childbirth and the postnatal period, the World Health Organization (WHO) proposes two complementary agendas: Strategies to end preventable maternal deaths and the Action Plan for All Newborns, both articulated to the new Global Strategy for Women, Children and Adolescent Health in the post-2015 Sustainable Development Objectives era^(^ ^1^ ^)^.

In Brazil, the quality care to the maternal-infant group is still a challenge and, therefore, issues such as early access to prenatal care and constant search for pregnant women without care, gestational risk identification, with integration of programs and activities in care networks and development of health education actions should be considered as a priority in primary health care^(^ ^2^ ^)^. In this sense, the Family Health is considered a new way of health work organization and a priority strategy for health services consolidation and expansion, without breaking completely with the traditional model, but seeking to improve the practices, improving the care actions and the determinants of morbidity and mortality^(^ ^3^ ^)^. The link between professional and patient is emphasized, having actions that enable to know the particular reality of each individual and family, which is one of the tools for its consolidation^(^ ^4^ ^–^ ^5^ ^)^.

The Basic Health Units of the Family Health Strategy (BHU-FS) rely on teams composed of doctors, nurses, nursing assistants or technicians, and community health agents, favoring the link between professionals and patients, responsibility of the teams and enabling continuity of care. The Basic Health Units of the traditional model (BHU-T) tend to keep professionals strongly guided by the biomedical and curative model, centered on the individual and with teams according to the characteristics and needs of each municipality. The presence of the professional community health agent is not required in the minimum structuring of the traditional model teams, differently to the recommendation of the Family Health Strategy teams. In both care models, the professionals work with patients´ ascription in a delimited area^(^ ^6^ ^)^.

Both models of care: BHU-FS and BHU-T develop actions directed to the maternal-infant group.

Specifically regarding to the prenatal care, a study conducted in southern Brazil comparing the traditional models and the Family Health Strategy found more guidelines on breastfeeding, postpartum contraception, puerperal consultation, care of the newborn, type of delivery and on the test for the detection of Human Immunodeficiency Virus (HIV) in the Family Health Strategy. Also, the pregnant women seen in the Strategy had more frequently their breasts examined and procedures such as blood pressure and uterine height verification performed, reinforcing the importance of this model for women's health care^(^ ^7^ ^)^.

In the city of Botucatu/SP, where this study was developed, BHU-T and BHU-FS models coexist. Comparing them to the quality of prenatal care, a research published in 2013, with data of 2010, indicated a similarity in the structure and more favorable indicators-synthesis of processes in the BHU-FS: set of exams recommended for the first and the third trimester (OR=6.68, CI=3.78-11.87); six consultations and a set of exams (OR=4.26, CI=2.46-7.39) and nutritional orientation and the warning signs in the term (OR=12.26; CI=6.36-23.94). There was also an advantage for BHU-FS when considering the set of recommended activities, consisted of six consultations, set of exams, tetanus vaccine and the puerperal consultation (OR=4.02, CI=2.04 -8.01)^(^ ^8^ ^)^.

Several Brazilian studies evaluate prenatal care based on the activities developed, or the care process^(^ ^7^ ^,^ ^9^ ^–^ ^10^). However, no research has been identified to evaluate the results of this care seeking relationships with indicators of the first year of life and to consider the different models of care. Thus, this study aimed to evaluate the process and outcome indicators of prenatal care developed in primary care, comparing traditional care models and the Family Health Strategy.

## Method

This is a prospective cohort study, aimed at the evaluation of prenatal care. The data source was the Botucatu-CLaB Infant Cohort study, whose objective was to know data, events, and situations related to the health of children living in that municipality, during their first year of life.

The study was developed in a municipality located in the center-south region of the state of São Paulo, with an estimated population of 144,820 inhabitants^(^ ^11^ ^)^ in 2018. It belongs to the Regional Health Department VI (DRS VI), Bauru, with 67 other municipalities. There are eight BHU-T and 12 BHU-FS with fifteen teams to attend prenatal care at the usual risk in the Unified Health System. The pathological prenatal follow-up is carried out in a Teaching Hospital that is a reference to other municipalities of the DRS VI and the only public service for attending childbirth, both at risk and at high risk, in the city of Botucatu.

The inclusion criteria in the cohort study were: being a newborn mother, living in Botucatu and being able to respond to interviews. The recruitment was carried out from July 27, 2015, to February 2, 2016, in a basic neonatal screening service, responsible for attending all the newborns of the municipality in the first month of life, regardless of whether the birth has occurred in the public or private service. The data sources in the cohort were: an interview with the mother, record of attendance in the neonatal screening service, the pregnant woman's card and the child's book. Data were collected to characterize the participants, regarding the process of prenatal care, delivery, and birth. The cohort was followed in six other moments to collect data regarding breastfeeding and introduction of complementary feeding: at two and four months, from telephone interview and at three, six, nine and 12 months of the child's life, per home visit. The end of follow-up was in February 2017.

**Figure 1:**
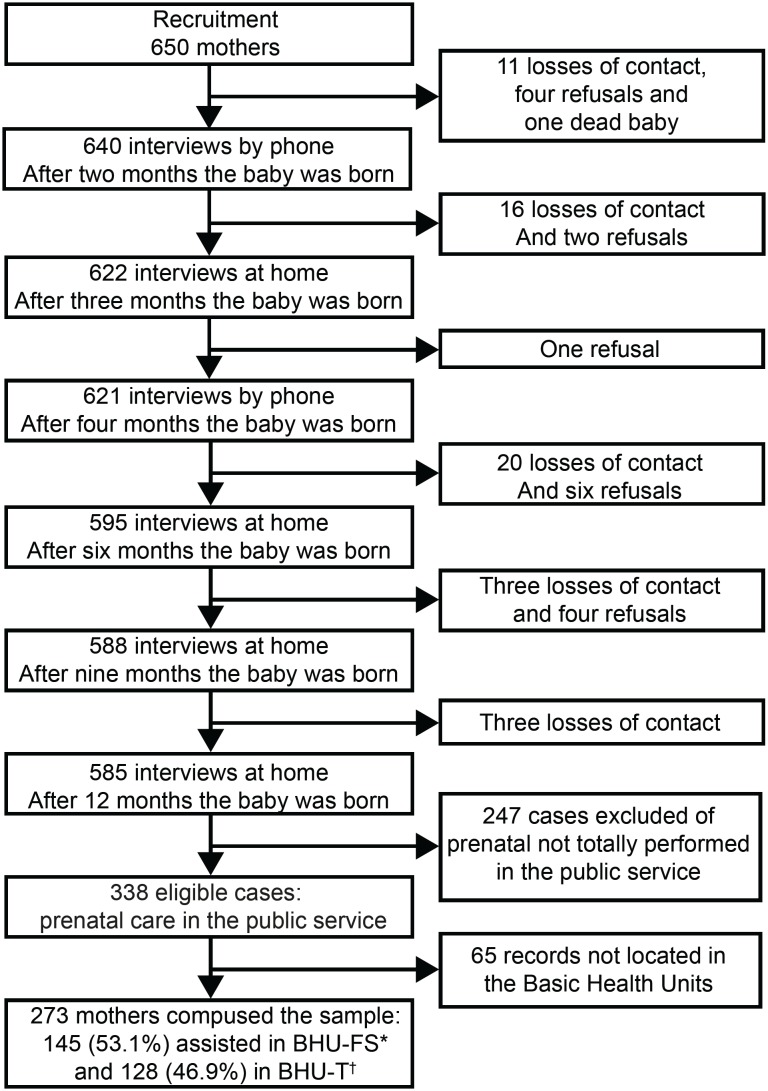
Flowchart of the cohort formation and the intentional sample composition of this study. Botucatu, 2015-2017 *BHU-FS = Basic Health Units of the Family Health Strategy; †BHU-T = Basic Health Units of the traditional model

There was a total of 650 mothers in the cohort. During the follow-up, there were 65 losses/refusals (10%), resulting in 585 binomials (mothers and babies) followed up to the 12^th^ month of life, of which 338 cases were eligible for this study because they were followed up exclusively in the public service during the prenatal. An intentional sample consisted of 273 mothers, whose records were located in the Basic Health Units (Figure 1).

The proportions intentionally obtained of 128 binomial mothers/infants (46.9%) assisted in BHU-T and 145 (53.1%) in BHU-FS, are similar to those found when considering the location of follow-up of Botucatu in 2017: 48.4% in BHU-T and 51.6% in BHU-FS^(^ ^12^ ^)^. The place where the recruitment took place assists women from all the basic health units of the municipality.

All the instruments used in the data collection were constructed specifically for this study and tested on 12 puerperal women not included in the sample, to adjust the questions that could have difficulties. Data collection was performed by a properly trained and remunerated team supervised by one of the authors of this study. The integrity of the interviews was verified by phone, in a random sample of 5% of the participants, through re-interviews performed by the field supervisor, also responsible for checking for inconsistencies and correction of the database.

The data obtained were discussed based on the theoretical reference of the social determinants of health, whose studies began in the 1970s to subsidize the understanding of the social relationships in the health-disease process and the causality of health problems. In this perspective, to analyze the health services and extending care coverage assists in improving the quality of care provided^(^ ^13^ ^)^.

The methodological reference of the evaluation proposed in the 1980s by Donabedian^(^ ^14^ ^)^ was adopted, specifically regarding the process components and outcomes. For this author, the study of the process has actions of health care, including diagnosis, treatment, preventive care, and health education, and therefore, its measurement is almost equivalent to the measurement of quality of care. The outcomes have the effects of health care on people or populations, and changes in their state of health.

Thus, an analysis of quality indicators of the prenatal care process is shown, according to the health care model and the effect on early health indicators (low birth weight, breastfeeding in the first hour of life, need for hospitalization in the Unit of Intensive Care or Neonatal Intermediate Care and intercurrence of labor at discharge) and late health indicators (exclusive breastfeeding at six months and breastfeeding at 12 months).

The independent variable (exposure) was a prenatal care model, both for process and outcome analysis, categorized in BHU-T and BHU-FS.

For the analysis of the process indicators, the dependent variables were selected based on the recommendations of the Brazilian Ministry of Health and addressed in other prenatal quality assessment studies^(^ ^7^ ^,^ ^15^ ^–^ ^17^ ^)^ and obtained from the data from the prenatal record and care records in Primary Care (yes, no): beginning up to 12 weeks; at least six consultations; (hemoglobin and hematocrit, fasting blood glucose, serology: syphilis, HIV, hepatitis B and toxoplasmosis, simple urine, urine culture, and blood typing); first trimester ultrasonography; (hemoglobin and hematocrit, fasting blood glucose, serology: syphilis, HIV and, if necessary, hepatitis B and toxoplasmosis, simple urine and uroculture); health education (health education was considered when the mother reported having been instructed on feeding, physical activity, warning signs at term and type of delivery) and puerperal consultation (performed up to 42 days after the postpartum).

For each yes answer (best situation), a score was assigned. Thus, these seven variables used to evaluate the quality of the prenatal process allowed the construction of a score, which ranged from zero (worse situation) to seven points (better situation). The score was considered low when equal or less than three points and this value was established after evaluating the mean and median scores of the group: 3.1 and 3, respectively.

For the outcome analysis, as proposed by Donabedian^(^ ^14^ ^)^, the dependent variables (outcome) were: low birth weight (yes, no); intercurrences in childbirth (yes, no); absence of breastfeeding in the first hour of life (yes, no); need for hospitalization in the Intensive Care Unit (ICU)/Intermediate Care Unit (IMCU) after birth (yes, no); intercurrences with the newborn from birth to discharge (yes, no); absence of exclusive breastfeeding at six months (yes, no) and weaning at 12 months (yes, no). The indicators related to the birth were obtained from the records of the children in the neonatal service and the baby's book, and the others, during an interview with the mothers.

As confounders, the following variables were included: maternal age at delivery in years (up to 19, 20 or more); years of school approval (up to eight, nine or more); skin color (not white, white); companion presence (yes, no); paid maternal work (yes, no); family per capita income less or equal to half a minimum wage (yes, no); primigestation (yes, no); gestation accepted by the mother (yes, no); intercurrence during pregnancy that required maternity care (yes, no); (yes, no) and type of delivery (vaginal, cesarean). All these variables were obtained from the interview with the mother. To identify the family per capita income, the family's income in reais was inquired, divided by the number of people dependent on that income, and there was a classification considering the value of Brazil's Minimum Wage in 2016.

The mean and median prenatal quality scores, with the respective standard deviations (SD) and minimum (min) and maximum (max) values, respectively, were identified according to the care model and compared by the Mann-Whitney test. The analysis of potential confounders and prenatal process indicators was performed by relative risk (RR) estimation. Then, the risk of occurrence of newborn events that express the health care outcome, as proposed by Donabedian^(^ ^14^ ^)^ according to the care model, was analyzed by estimating RR by Cox Multiple Regression Model, adjusted for potential confounders (p<0.20). Relationships were considered significant if p<0.05. The analyses were performed with Statistical Package for the Social Sciences (SPSS) software version 21.0.

The study was approved by the Research Ethics Committee of the Botucatu Medical School of the “Júlio de Mesquita Filho” State University under Certificate 7628714.8.0000.5411.

## Results

Women who underwent prenatal care in the BHU-FS had a significantly higher risk of per capita income equal or lower than 0.5 MW (RR=1.52, 95% CI=1.04-2.21) when compared to those with a prenatal in the BHU-T (Table 1).

**Table 1:** Maternal socio-demographic, prenatal and childbirth characteristics, according to the health care model. Botucatu, SP, Brazil, 2015-2017

Variables		BHU-T* (N=128)		BHU-FS† (N=145)		RR‡ (CI95%)§
		N	%	N	%	
Age in years						
	Up to 19	23	17.9	34	23.4	1.30 (0.77-2.21)
	20 or more	105	82.1	111	76.6	1
Years of school approval						
	Up to 8	27	21.1	44	30.3	1.44 (0.89-2.32)
	9 or more	101	78.9	101	69.7	1
Skin color						
	Non-white	59	46.1	69	47.6	1.03 (0.73-1.46)
	White	69	53.9	76	52.4	1
Partner						
	No	24	18.7	23	15.9	0.84 (0.48-1.50)
	Yes	104	81.3	122	84.1	1
Paid work						
	No	65	50.8	91	62.8	1.23 (0.90-1.70)
	Yes	63	49.2	54	37.2	1
Income ≤ 0,5 Mw‖ Per Capita						
	Yes	43	33.6	74	51.0	1.52 (1.04-2.21)
	No	85	66.4	71	49.0	1
Primigestation						
	No	69	53.9	84	57.9	1.07 (0.78-1.48)
	Yes	59	46.1	61	42.1	1
Gestation accepted						
	No	20	15.6	12	8.3	0.53 (0.26-1.08)
	Yes	108	84.4	133	91.7	1
Prenatal Intercurrence						
	Yes	85	66.4	83	57.2	0.86 (0.63-1.16)
	No	43	33.6	62	42.8	1
Intercurrence in childbirth						
	Yes	23	17.9	20	13.8	0.77 (0.42-1.40)
	No	105	82.1	125	86.2	1
Type of birth						
	Cesarean section	43	33.6	47	32.4	0.96 (0.64-1.46)
	Vaginal	85	66.4	98	67.6	1

* BHU-T = Basic Health Units of the traditional model;

† BHU-FS = Basic Health Units of the Family Health Strategy;

‡ RR = Relative Risk;

§ CI 95% = Confidence Interval of 95%;

‖ Mw = minimum wage in Brazil, value R$ 880.00 on 01/01/2016

The risk of the low quality score was lower in the BHU-FS (RR=0.70, CI95%=0.52-0.95), while the risk of postnatal consultation (RR=1.48, CI95%=1.08-2.04) and to receive health education (RR=13.70, CI95%=3.27-57.17) was higher in these Units when compared to the BHU-T (Table 2).

**Table 2:** Prenatal care process, based on the quality score and variables that compose it, according to the health care model. Botucatu, SP, Brazil, 2015-2017

Variables		BHU-T* (N=128)		BHU-FS† (N=145)		RR‡ (CI95%)§
		N	%	N	%	
Early start (up to 12 weeks)						
	Yes	76	59.4	92	63.4	1.07 (0.79-1.45)
	No	52	40.6	53	36.6	1
At least six consultations						
	Yes	102	79.7	124	85.5	1.07 (0.82-1.39)
	No	26	20.3	21	14.5	1
First trimester examinations						
	Yes	48	37.5	66	45.5	1.21 (0.83-1.76)
	No	80	62.5	79	54.5	1
Ultrasound in the first trimester						
	Yes	27	21.1	47	32.4	1.54 (0.96-2.48)
	No	101	78.9	98	67.6	1
Third Trimester Examinations						
	Yes	32	25.0	51	35.2	1.40 (0.90-2.19)
	No	96	75.0	94	64.8	1
Health education						
	Yes	39	30.5	104	71.7	13.70 (3.27-57.17)
	No	89	69.5	41	28.3	1
Postpartum consultation						
	Yes	60	46.9	101	69.7	1.48 (1.08-2.04)
	No	68	53.1	44	30.3	1
A low score (≤3)						
	Yes	93	72.7	74	51.0	0.70 (0.52-0.95)
	No	35	27.3	71	49.0	1

* BHU-T = Basic Health Units of the traditional model;

† UBHU-FS = Basic Health Units of the Family Health Strategy;

‡ RR = Relative Risk;

§ CI 95% = Confidence Interval of 95%

The median prenatal quality score for the two care models was 3.0, ranging from 0-6 to 0-7 for BHU-T and BHU-FS. The mean score was significantly higher (p<0.001) in the BHU-FS (3.53, SD = 1.50) than in the BHU-T (2.71, SD = 1.33) (data not shown in the table).

When there were confounders, there was no difference between BHU-T and BHU-FS for the early indicators studied: school approval equal or less than 8 years, paid maternal work and family per capita income equal or less than 0.5 minimum wages (Table 3).

**Table 3:** Multiple regression of Cox for the indicators of early outcomes, according to the model of care (BHU-T/BHU-FS)*. Botucatu, SP, Brazil 2015-2017

RR† (CI95%)‡				
Early Indicators	Low weight at birth	The absence of breastfeeding in the first hour of life	Admission ICU/IMCU§	Birth intercurrence at discharge
BHU-T/BHU-FS*	0.89(0.23-3.45)	0.77(0.47-1.26)	0.63(0.29-1.36)	1.07(0.69-1.66)
School Approval	1.32(0.30-5.68)	1.03(0.57-1.84)	1.11(0.48-2.60)	0.99(0.60-1.64)
Paid work	0.74(0.18-3.06)	0.87(0.52-1.45)	1.07(0.48-2.35)	1.10(0.69-1.73)
Income < 0.5 MW‖ PC¶	1.88(0.45-7.82)	0.70(0.41-1.21)	1.07(0.48-2.36)	0.88(0.55-1.40)
Gestation accepted	0.0(0.0-0.0)	1.03(0.49-2.18)	1.80(0.72-4.50)	1.27(0.68-2.37)

* BHU-T/BHU-FS = Basic Health Units of the Traditional Model/Basic Health Units of the Family Health Strategy;

† RR = Relative Risk;

‡ CI 95% = Confidence Interval of 95%;

§ ICU/IMCU = Intensive Care Unit/Intermediate Care Unit;

‖ MW = minimum wage in Brazil, value R$ 880.00 on 01/01/2016;

¶ PC = Per Capita

There was also no difference between BHU-T and BHU-FS for the late indicators when considering the confounders: school approval equal or less than 8 years, paid maternal work and family per capita income equal or less than 0.5 minimum wages (Table 4).

**Table 4:** Multiple Cox regression for late outcome indicators, according to the model of care (BHU-T/BHU-FS)*. Botucatu, 2015-2017

RR† (IC95%)‡		
Late Indicators	Absence of EBF§ in the sixth month Of life	Weaning 12 months of life
BHU-T/BHU-FS *	0.99 (0.73-1.33)	0.90 (0.64-1.25)
School Approval	1.01 (0.71-1.43)	0.93 (0.62-1.38)
Paid work	0.85 (0.62-1.15)	0.97 (0.69-1.37)
Income < 0.5 MW‖ Per Capita	1.12 (0.82-1.53)	0.92 (0.64-1.31)
Gestation accepted	1.09 (0.71-1.68)	1.20 (0.74-1.94)

* BHU-T/BHU-FS = Basic Health Units of the Traditional Model/Basic Health Units of the Family Health Strategy;

† RR = Relative Risk;

‡ CI 95% = Confidence Interval of 95%;

§ EBF = Exclusive Breastfeeding;

‖ MW = minimum wage in Brazil, value R$ 880.00 on 01/01/2016

## Discussion

The analysis of the indicators of the prenatal care process, based on the created score, showed a better situation for women attending the BHU-FS. However, there was no difference between the models of primary care in the outcome of care, both when considering the early and late indicators.

Also, it was found that the women attending the BHU-FS had worse socioeconomic conditions, since they were more frequently classified in the lower income group and, therefore, they were expected to have worse indicators.

It is already known that the health-disease is produced and distributed in society through strong processes of social, economic, cultural, environmental, political determination, among others. At the global level, there are also recently initiatives aimed at fostering policies inspired by the Social Determinants of Health, stating that economic and social conditions decisively influence the health conditions of people and populations, although not all the determinants are equally important. Thus, those that generate social stratification, called structural determinants are highlighted, with the distribution of income among them^(^ ^18^ ^)^.

Thus, worse indicators were expected in the BHU-FS group, due to the worse income condition, which did not occur. A possible interpretation for the findings of this study are from the fact that these women received more qualified prenatal care, and this condition may have minimized the unfavorable effect of poverty.

However, a study on prenatal care performed in the Unified Health System (SUS), in a micro-region of Espírito Santo, according to the procedures proposed by the Brazilian Ministry of Health, assessed this assistance as inadequate, especially in women of lower income and patients of the Program of Community Health Agents^(^ ^19^ ^)^.

In the isolated approach of the variables with the quality score, a better performance of the BHU-FS in health education actions is observed. In this context, the differential role of nurses has been emphasized, since it has acted from raising awareness about the importance of follow-up in this period, understanding the meaning of this moment in the pregnant woman's life^(^ ^20^ ^)^. A recent study carried out in Portugal corroborates it since more than half of the mothers who were considered to have a higher level of knowledge reported being the primary source of information during prenatal care^(^ ^21^ ^)^.

Theoretical research reporting that the Family Health Strategy is a technological innovation in the health area, in the work carried out, includes the assistance accomplished, adding multiple dimensions, such as the educational actions, which highlights the importance of its performance^(^ ^3^ ^)^. Regarding these actions, life habits such as physical activity and healthy eating, as well as important aspects related to childbirth and birth, such as warning signs at the end of pregnancy and type of delivery were approached in the present study. The best result found, in which the women attending the BHU-FS received more guidance during the prenatal period than those attended by the BHU-T (RR = 13.70, CI = 3.27-57.17), was also observed in a similar study carried out in southern Brazil in 2012^(^ ^7^ ^)^. On the other hand, a study on the practice of health education in BHU-FS pointed out that the academic training received by the professionals of these units, in general, is insufficient to subsidize them in the effective work with the community, being an obstacle to overcome^(^ ^22^ ^)^.

In both care models, the coverage of the puerperium consultation was low, although it was significantly higher in the BHU-FS (69.7%) than in the BHU-T (46.9%). In the same municipality, a study in 2012^(^ ^8^ ^)^ indicated that 58.2% of the women who underwent prenatal care at the BHU-FS returned to the health service after the child was born for puerperal consultation. Thus, although still far from ideal, there was an improvement in the coverage of the puerperium consultation in 11.5% in the BHU-FS of Botucatu. No data on puerperal consultation were found in other studies that evaluated prenatal care according to the care model^(^ ^7^ ^,^ ^23^ ^)^.

Regarding early outcome indicators, unlike the findings of this research, a national study reveals that not having performed prenatal care adequately, that is, poor quality prenatal follow-up, it has decreased the probability of breastfeeding in the first hour of life^(^ ^24^ ^)^, and in another study, there was a significant correlation between ICU stay and prenatal care, and the children of women who did not perform prenatal care were hospitalized for longer. This last research, corroborating with the findings of this study, found no association between intercurrences at birth and prenatal follow-up^(^ ^25^ ^)^.

Low birth weight as another indicator of early outcome adopted, was addressed in a study on prenatal quality developed at BHU-FS in the metropolitan region of Campina Grande, Paraíba. The conclusion also suggests that adequate prenatal care may have mitigated the influence of socioeconomic inequalities related to health care^(^ ^26^ ^)^.

Regarding the late indicators, regardless of the care model, research developed in Canada did not show a relationship between prenatal quality and duration of breastfeeding in the first six months of the baby^(^ ^27^ ^)^, findings that corroborate this research. As for weaning, a study in southwestern Michigan found that women who started prenatal care in the first trimester of pregnancy were more likely to continue breastfeeding than those who started prenatal follow-up belatedly, highlighting the importance of follow-up in prenatal care^(^ ^28^ ^)^.

This study was developed in a medium-sized municipality in the interior of São Paulo, and it is possible that the results obtained are also found in other municipalities with similar characteristics. This, the fact that part of the data was obtained from the records of attendance of the children in the neonatal service, the card of the pregnant woman and the baby's book is a limitation to be considered. Thus, in any study in which data collection is dependent on the registry of professionals, which was not registered, it was considered not performed.

## Conclusion

The mothers attended at the BHU-FS had worse socioeconomic status and the evaluation of the prenatal care process was more favorable in this group.

There was no difference between BHU-T and BHU-FS in the evaluation of outcome indicators, despite the large breadth of the indicators analyzed. Possibly the best quality of prenatal care was able to minimize negative socioeconomic effects, so the evaluation of outcome indicators was similar between the two models of health care.
